# The genome analysis of *Oleiphilus messinensis* ME102 (DSM 13489^T^) reveals backgrounds of its obligate alkane-devouring marine lifestyle

**DOI:** 10.1016/j.margen.2017.07.005

**Published:** 2017-12

**Authors:** Stepan V. Toshchakov, Alexei A. Korzhenkov, Tatyana N. Chernikova, Manuel Ferrer, Olga V. Golyshina, Michail M. Yakimov, Peter N. Golyshin

**Affiliations:** a Immanuel Kant Baltic Federal University, 236040 Kaliningrad, Russia; b School of Biological Sciences, Bangor University, LL57 2UW Bangor, Gwynedd, UK; c Institute of Catalysis CSIC, Campus Cantoblanco, 28049 Madrid, Spain; d Institute for Coastal Marine Environment, CNR, 98122 Messina, Italy

**Keywords:** *Oceanospirillales*, Marine alkane-degrading bacteria, Hydrocarbonoclastic, *Oleiphilus messinensis*

## Abstract

Marine bacterium *Oleiphilus messinensis* ME102 (DSM 13489^T^) isolated from the sediments of the harbor of Messina (Italy) is a member of the order *Oceanospirillales*, class *Gammaproteobacteria*, representing the physiological group of marine obligate hydrocarbonoclastic bacteria (OHCB) alongside the members of the genera *Alcanivorax*, *Oleispira*, *Thalassolituus*, *Cycloclasticus* and *Neptunomonas*. These organisms play a crucial role in the natural environmental cleanup in marine systems. Despite having the largest genome (6.379.281 bp) among OHCB, *O. messinensis* exhibits a very narrow substrate profile. The alkane metabolism is pre-determined by three loci encoding for two P450 family monooxygenases, one of which formed a cassette with ferredoxin and alcohol dehydrogenase encoding genes and alkane monoxygenase (AlkB) gene clustered with two genes for rubredoxins and NAD^+^-dependent rubredoxin reductase. Its genome contains the largest numbers of genomic islands (15) and mobile genetic elements (140), as compared with more streamlined genomes of its OHCB counterparts. Among hydrocarbon-degrading *Oceanospirillales*, *O. messinensis* encodes the largest array of proteins involved in the signal transduction for sensing and responding to the environmental stimuli (345 *vs* 170 in *Oleispira antarctica*, the bacterium with the second highest number). This must be an important trait to adapt to the conditions in marine sediments with a high physico-chemical patchiness and heterogeneity as compared to those in the water column.

## 1 Introduction

**Fig. 1:**
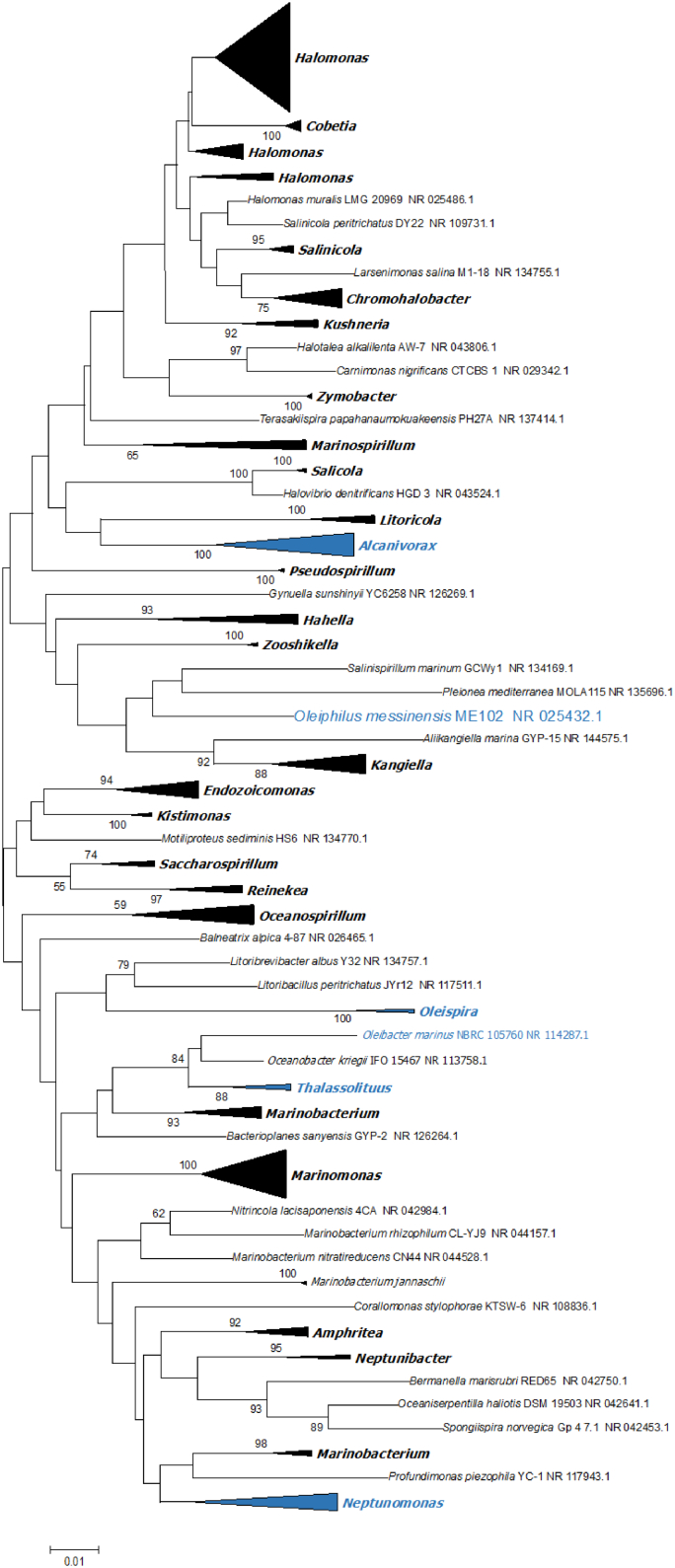
Phylogenetic position of *O. messinensis* ME102 and other OHCB (marked in blue) among 250 species of *Oceanospirillales*, as revealed by SSU rRNA gene sequence analysis. The evolutionary relationships were inferred using the Neighbor-Joining method (Saitou and Nei, 1987). The optimal tree with the sum of branch length = 3.41994391 is shown. The percentage of replicate trees in which the associated taxa clustered together in the bootstrap test > 50% (1000 replicates) are shown next to the branches (Felsenstein, 1985). The tree is drawn to scale, with branch lengths in the same units as those of the evolutionary distances used to infer the phylogenetic tree. The evolutionary distances were computed using the Jukes-Cantor method (Jukes and Cantor, 1969) and are in the units of the number of base substitutions per site. The rate variation among sites was modeled with a gamma distribution (shape parameter = 1). The analysis involved 251 nucleotide sequences. All positions containing gaps and missing data were eliminated. There were a total of 464 positions in the final dataset. Evolutionary analyses were conducted in MEGA6 (Tamura et al., 2013). (For interpretation of the references to colour in this figure legend, the reader is referred to the web version of this article.)

*Oleiphilus messinensis* is one of the organisms that represent the functional group of so-called obligate marine hydrocarbonoclastic bacteria (OHCB), which plays a pivotal role in the degradation of petroleum constituents in the sea (Yakimov et al., 2007). The strain *O. messinensis* ME102 (DSM 13489^T^) was isolated from the sediments of the Messina harbor, which is severely affected by the ferry traffic, after the enrichment of the sample with tetradecane in the artificial seawater medium ONR7a. *O. messinensis* was described to represent a new species within the new genus *Oleiphilus*, within the novel family *Oleiphilaceae* of *Oceanosprillales (Gammaproteobacteria)* (Golyshin et al., 2002). These Gram-negative aerobic bacteria have a very restricted substrate range, consistent with their OHCB designation, preferring aliphatic hydrocarbons, fatty acids and alcohols, as carbon and energy sources over sugars and amino acids (Golyshin et al., 2002). Fig. 1 depicts the placement of *O. messinensis* pointing at its relatively distant placement on the phylogenetic tree with other *Oceanospirillales*. Worth attention, the genus *Oleiphilus* is currently only represented by a single isolate (type strain) with another one to share 98% SSU rRNA sequence identity (GenBank Acc Nr FJ845394), pointing at a rather endemic nature of this particular species. This is in the stark contrast with other OHCBs that include very ubiquitous *Alcanivorax* spp. (Yakimov et al., 1998, Schneiker et al., 2006), *Oleispira* spp. important in polar and deep (cold) marine environments (Yakimov et al., 2003, Kube et al., 2013), *Thalassolituus* spp., an oil-degrader occupying various marine niches, including estuarine waters (Yakimov et al., 2004, McKew et al., 2007, Golyshin et al., 2013) and PAH-degrading specialists from the genus *Cycloclasticus* (Dyksterhouse et al., 1995, Geiselbrecht et al., 1998, Lai et al., 2012, Messina et al., 2016). Here, we report on the genome-based analysis of obligate marine hydrocarbonoclastic bacterium, *Oleiphilus messinensis* ME102. Genome analysis revealed exceptional genome plasticity of ME102, showing an unprecedented abundance of mobile elements for a member of the *Oceanospirillales*, which could potentially play an important role in the of genome regulatory circuits.

## 2 Results and Discussion

### 2.1 General features of *O. messinensis* genome

**Fig. 2:**
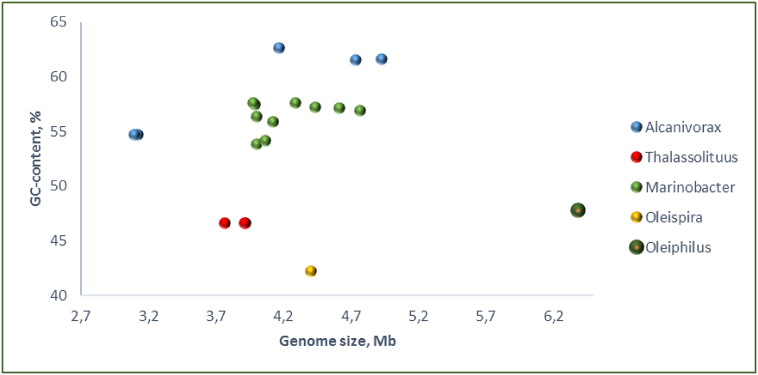
Genome size *vs.* GC-content in complete genomes of gammaproteobacterial genera with hydrocarbonoclastic representatives.

The genome of *O. messinensis* was sequenced using hybrid approach using Roche 454 and Illumina sequencing technologies. Assembly was performed with Newbler and Phred/Phrap/Consed *de novo* assemblers resulting in 6.38 Mb circular chromosome sequenced with 119 × read coverage. Genome size of *O. messinensis* is the largest among other hydrocarbonoclastic *Oceanospirillales* reported earlier (Fig. 2). Average GC content was 47.8%. Genome contains 5502 protein-coding genes, 4081 (74,1%) of which were assigned with function. Five complete ribosomal operons have identical structure: 16S – tRNA-Ile – tRNA-Ala – 23S – 5S. *O. messinensis* genome was significantly enriched with mobile elements, > 50% of which were active (Table 1). Analysis of bacteriophage-related genes with PHASTER (Arndt et al., 2016) revealed two putative prophage regions of 21.5 kb total.

**Table 1:** Genome and environmental features of *Oleiphilus messinensis*.

Feature	Description
Current classification	Domain *Bacteria* Phylum *Proteobacteria* Class *Gammaproteobacteria* Order *Oceanospirillales* Family *Oleiphilaceae* Genus *Oleiphilus* Species *Oleiphilus messinensis* Type strain ME102^T^
Biosample ref	SAMN06234459 (NCBI)
Collection date	21-Jun-1998
Geographical location name	Italy, Messina harbor, 38.19 N 15.56 E
Geographical coordinates	38.19 N 15.56 E
env_biome	Sea
env_feature	Harbor
env_material	Water/sediment
Depth, m	8
Biotic relationship	Free living
Relation to oxygen	Aerobe
Sequencing project ref	PRJNA362330 (NCBI)
Sequencing method	Roche 454, Illumina GAx
Sequencing center	Fidelity Systems
Assembly method	Newbler v. 2.6; Phred/Phrap/Consed v. 23.0
Coverage	119 ×
Number of replicons	1
Finishing level	Finished
Genome size, bp	6,379,281
GC content, %	47.8
Genes	5502
Pseudogenes	53
RNA genes	74
rRNA	15 (5 operons)
tRNA	59
GI number	15
GI length (% share)	218,392 (3.4)
Mobile elements	142
Complete ORFs	85
Partial ORFs	57
Number of different IS families	14
Prophage regions	2
Intact	–
Partial	2
Total length (kb)	21.5

### 2.2 Genome-based phylogenetic position

**Fig. 3:**
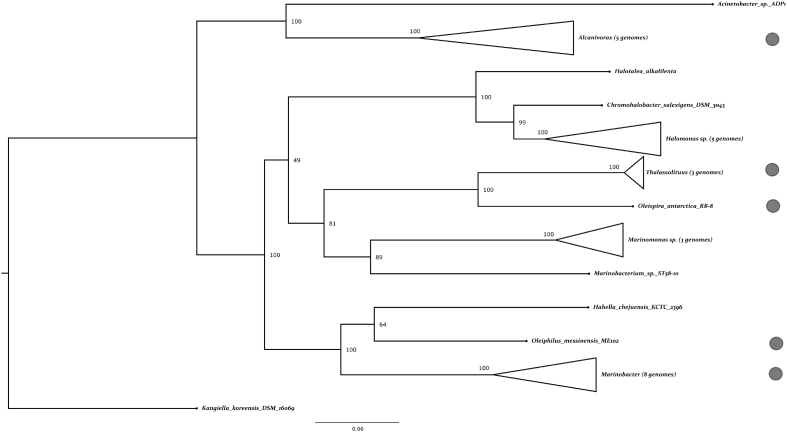
Maximum-likelihood phylogenetic tree of representatives of *Oceanospirillales* family based on concatenated alignment of 22 ribosomal proteins. For phylogenetic analysis, we took complete genomes of free-living gammaproteobacteria from *Oceanospirillales*, *Alteromonadales* (30 and 98 respectively) and *Acinetobacter* sp. ADP1 (order *Pseudomonadales*). Sequences of 22 ribosomal proteins for each genome were accessed using *hmmsearch* script from HMMER 3.1b2 package (e-value < 10^− 7^, query coverage > 70%) (Finn et al., 2015). 111 genomes possessing all queried ribosomal proteins were used in the further analysis. Sequences for each protein were aligned with Clustal Omega (ver. 1.2.1) and concatenated to one alignment (Sievers and Higgins, 2014). Sites having > 95% gaps were deleted. Phylogenetic tree was constructed using maximum likelihood method in RAxML (version 8.2.4) with following options: protein model - LG (best scoring), rate heterogeneity model - GAMMA, 100 bootstraps (Stamatakis, 2014). On the basis of initial tree analysis (Supplementary Fig. 2) *Kangiella* sp. was chosen as an outgroup. For the final tree construction 31 sequences of OHCB were realigned with Clustal Omega. Tree was constructed in RAxML using parameters described above. Lineages including OHCB marked with gray circles.

Phylogenetic reconstruction of marine hydrocarbonoclastic Gammaproteobacteria using ribosomal proteins showed that *O. messinensis* represents a deep lineage of *Oceanospirillales*. *Hahellaceae* family with the only sequenced genome of *Hahella chejuensis* KCTC 2396 (Jeong et al., 2005) was the closest relative to *Oleiphilus* spp. (Fig. 3). While 16S rRNA identity of ME102 and KCTC2396 was on the border of family threshold (92%), these microorganisms are characterized by quite different substrate preferences and other phenotypic characteristics (Lee et al., 2001, Golyshin et al., 2002). Genomic data also supports their classification as type strains of different families. In addition to significant GC difference (6.07%), mean two-way amino acid identity of two *in silico* proteomes was 53.17% (Supplementary Fig. 1) which is typical for inter-family comparisons (Konstantinidis & Tiedje, 2005).

Interestingly, the group of *Marinobacter* species, currently classified within the family *Alteromonadaceae* of *Alteromonadales*, formed monophyletic group, which was more closely related to *Oleiphilaceae*, than other hydrocarbonoclastic *Oceanospirillales*.

### 2.3 Alkane metabolism

As a typical representative of marine obligate hydrocarbonoclastic bacteria (OHCB) group, *O. messinensis* ME102^T^ grows preferentially on aliphatic hydrocarbons, alkanoates and alkanoles, as sole carbon and energy sources (Golyshin et al., 2002). Expectedly, its genome encodes many genes and operons presumably involved in hydrocarbon metabolism.

**Fig. 4:**
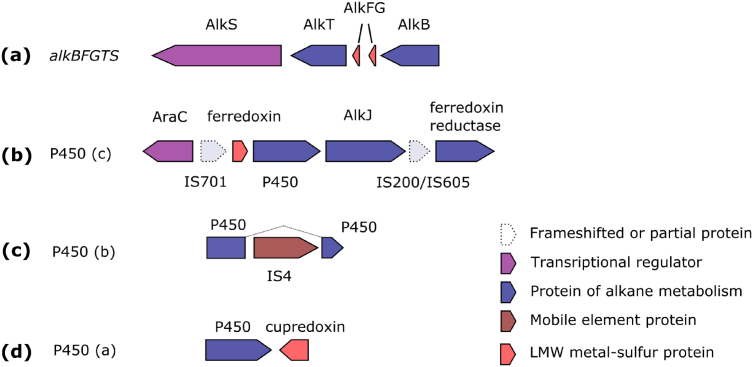
Schematic representation of gene clusters involved in alkane degradation in *O. messinensis*. See text and legend for details.

The first step of alkane utilization involves terminal hydroxylation of alkanes by alkane-1-monooxygenase. OLMES_3728 protein, orthologous to AlkB_1_ alkane hydroxylase of *A. borkumensis* SK2 (Schneiker et al., 2006) clusters with two rubredoxin genes (OLMES_3726 and OLMES_3727), NAD^+^-dependent rubredoxin reductase (OLMES_3725) and transcriptional regulator OLMES_3724 (Fig. 4a). OLMES_3724 belongs to LuxR family of transcriptional regulators and has a significant level of homology with AlkS regulator, known to activate transcription of *alk* operon in the presence of alkanes (Kok et al., 1989, Canosa et al., 2000). Thus, OLMES_3724 – OLMES_3728 *alkBFGTS* cluster forms a complete minimal set of genes necessary for primary oxidation of medium-chain alkanes. Another alkane-1-monooxygenase gene, presented in closely related to ME102^T^ OHCB *A. borkumensis* SK2, *alkB*_*2*_ was apparently lost during evolution.

*A. borkumensis* SK2 as well as other OHCB also involves P450-like cytochromes for the oxidation of medium-chain alkanes (Sabirova et al., 2011). *O. messinensis* ME102 harbors 3 loci, containing proteins, orthologous to P450 of *A. borkumensis*, known to be upregulated in the presence of isoprenoid hydrocarbons (Schneiker et al., 2006). P450(c)-like protein OLMES_4335 clusters with AraC transcriptional regulator (OLMES_4332), ferredoxin (OLMES_4334) and OLMES_4336 alcohol dehydrogenase, similar to AlkJ alcohol (alkanol) dehydrogenase. P450(b)-like protein seems to be disrupted by the insertion of IS4 family IS element, which, considering the completeness of transposase gene and inverted repeats happened recently (Fig. 4c). OLMES_5166 protein, orthologous to P450(a) was found to be isolated from other genes involved in alkane metabolism, but positioned next to OLMES_5167 cupredoxin. Decreased diversity of *alkB* genes and P450 cytochromes can explain narrower spectra of hydrocarbons, which can be utilized by *O. messinensis*, as compared to *A. borkumensis* (Golyshin et al., 2002, Yakimov et al., 1998).

Hydroxylation of long-chain alkanes (18 carbon atoms and longer) requires other alkane hydroxylation systems, involving AlmA (Throne-Holst et al., 2007) or LadA (Wentzel et al., 2007) hydroxylases. ME102^T^ is able to utilize alkanes up to 20 carbon atoms long (Golyshin et al., 2013). That phenotype may be explained by the fact that ME102^T^ contains FAD-binding monooxygenase OLMES_5009, having high level of homology (54% amino acid identity) with AlmA long-chain alkane monooxygenase of *Acinetobacter*.

Further steps in alkane catabolism involve successive alkanol oxidation steps resulting in acyl-CoA, which then metabolized through beta-oxidation. In experimentally characterized *Pseudomonas putida alk* operon it involves AlkJ alkanol dehydrogenase, AlkH aldehyde dehydrogenase and AlkK fatty-acid CoA ligase (van Beilen et al., 2001). In *O. messinensis* these genes were not clustered around central *alkBFGTS* operon, but rather scattered across the genome. Thus, putative AlkJ alcohol (alkanol) dehydrogenase OLMES_4336 is located near P450 cytochrome. ME102 possess several NAD-dependent aldehyde dehydrogenases, which can be involved in alkane-derived aldehyde oxidation. OLMES_0331 medium-chain-fatty-acid CoA ligase can act as AlkK protein, providing acyl-CoA for further beta-oxidation. As it was previously reported, majority of above gene clusters in *O. messinensis* are situated on horizontally acquired “catabolic transposons”, which is a very common phenomenon in marine oil-degrading bacteria (Yakimov et al., 2007).

Genome analysis also revealed coding potential of *O. messinensis* for the utilization of other hydrocarbon derivatives. OLMES_3894 haloalkane-dehalogenase-like protein, which does not have orthologs in other OHCB can possibly convert 1-haloalkanes to primary alcohol. Despite its activity requires further experimental validation, it can be inferred that this enzyme might determine a biotechnological potential of ME102^T^ in bioremediation of toxic environmental pollutants.

### 2.4 Genomic islands and mobile genetic elements

**Fig. 5:**
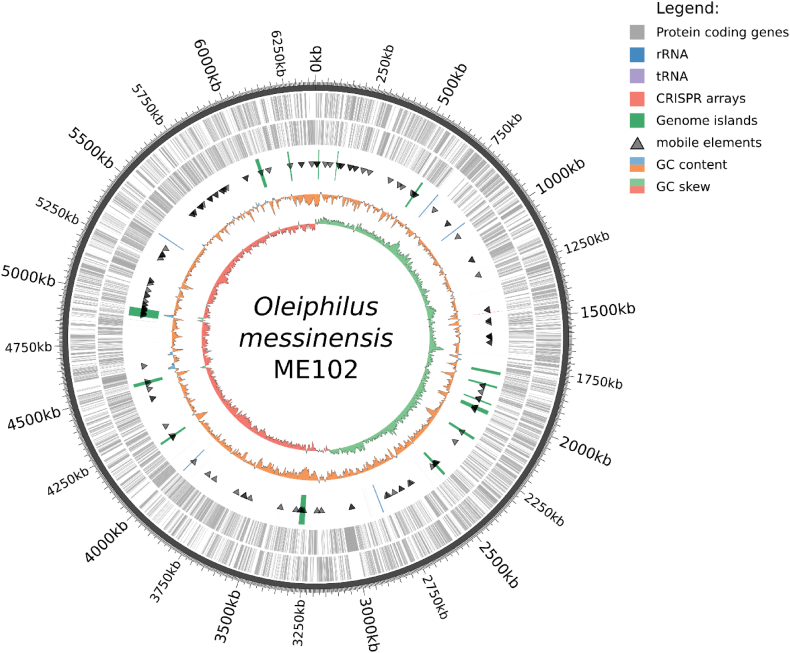
The genome map of *O. messinensis* ME102.

Genome of *O. messinensis* contains 15 genomic islands (GIs), most of which are associated with active mobile-element related genes (Fig. 5). Total length of all predicted GI was 218,392 bp, equivalent to 3.4% of the genome. Analysis of genes, associated with genomic islands, allowed to find out that most of fixed HGT events are of adaptive nature. Most of identified GIs contains genes involved in adaptation to environmental stimuli in marine environments. 9 of 15 Gis contain genetic determinants for bacterial defense and competition systems, such as RelB/RelE (OLMES_0561-OLMES_0562; OLMES_4307-OLMES_4308), VapB/VapC (OLMES_3971-OLMES_3972; OLMES_4311-OLMES_4312), MazE/MazF (OLMES_5329-OLMES_5330), Phd-Doc (OLMES_3985-OLMES_3986) toxin-antitoxin systems; type II (OLMES_5494-OLMES_5495) and type III (OLMES_3674-OLMES_3675) restriction-modification systems. GI4 contains at least two Rhs family proteins, known to be involved in inter- and intraspecies competition(Murdoch et al., 2011).

The large island (GI10) spanning 30.5 kbp contains several genes of widespread bacterial chemotaxis operon (OLMES_2869-OLMES_2876) including CheA histidine kinase, CheY and CheW response regulators and methyl-accepting chemotaxis protein MCP (Wuichet et al., 2007) as well as some genes required for mercury fitness, OLMES_2880 – OLMES_2882 (Sone et al., 2013).

The biggest GI identified in *O. messinesis* genome, the GI13, spanning over 31.7 kbp, possesses genes that might be essential for the phenotype of the organism. This GI contains cytochrome P450 alkane hydroxylase, OLMES_4335 involved in terminal hydroxylation of alkane hydrocarbons, located next to alcohol dehydrogenase (OLMES_4336), which might play a role in further steps of alkane utilization.

*O. messinensis* genome harbors 142 functional and inactivated mobile elements, occupying totally over 192 kbp. Analysis of transposase diversity performed with ISSaga (Varani et al., 2011) showed that they belong to at least 14 different families (Supplementary Table 1) which is rather exceptional among other *Oceanospirillales*, typically possessing 1–4 different IS families. Search for triggers of such genome plasticity among *Oceanospirillales* didn't reveal any significant result, therefore we suggest that expansion of mobile genetic elements might be beneficial to adaptation of changeable marine environments not only by direct transfer of metabolically important genes, but also by attenuation of transcriptional activity of adjacent genes by action of outward-oriented promoters, carried by certain type of IS elements (Vandecraen et al., 2017). IS1380-related elements which seemed to be most active in ME102 genome represented by 18 complete transposase ORFs, known to modulate the expression of genes involved in metronidazole (Soki et al., 2006), carbapenem (Kato et al., 2003) and blasticidine resistance (Lartigue et al., 2006). IS3 elements, also widespread and active in ME102 (17 functional copies), are shown to activate genes involved in acetate (Treves et al., 1998) or citrate utilization (Blount et al., 2012).

### 2.5 *Oleiphilus messinensis* has a distinguished standing among oil-degrading *Oceanospirillales*

**Fig. 6:**
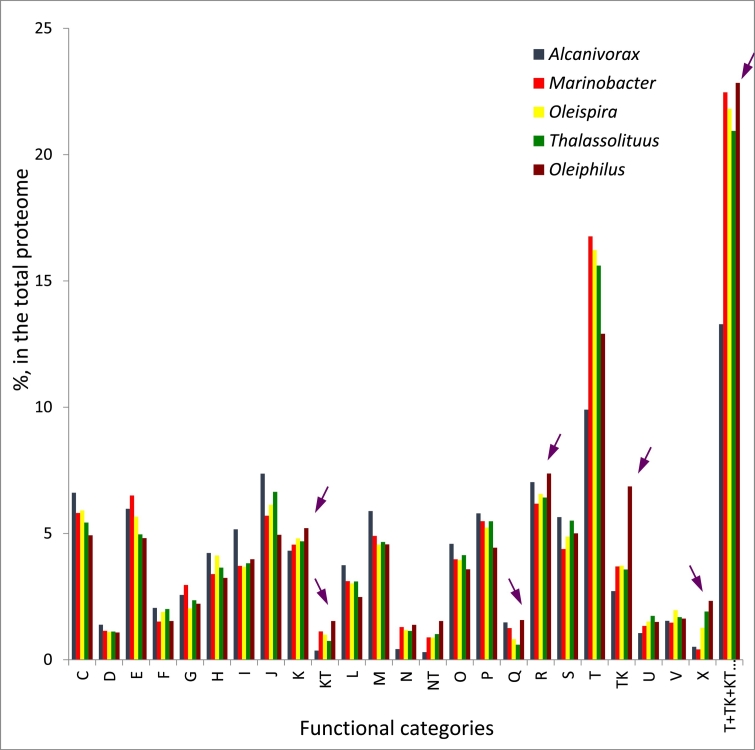
Distribution of proteins per functional categories of Clusters of Orthologous Groups of proteins (COGs) in genomes of the most significant marine oil degraders. The functional classification of the COGs is shown following functional categories: C, Energy production and conversion; D, Cell cycle control, cell division, chromosome partitioning; E, Amino acid transport and metabolism; F, Nucleotide transport and metabolism; G, Carbohydrate transport and metabolism; H, Coenzyme transport and metabolism; I, Lipid transport and metabolism; J, Translation, ribosomal structure and biogenesis; K, Transcription; L, Replication, recombination and repair; M, Cell wall/membrane biogenesis; N, Cell motility; O, Posttranslational modification, protein turnover, chaperones; P, Inorganic ion transport and metabolism; Q, Secondary metabolites biosynthesis, transport and catabolism; R, General function prediction only; S, Function unknown; T, Signal transduction mechanisms; U, Intracellular trafficking and secretion; V, Defense mechanisms (Galperin et al., 2015). Purple arrowheads indicate functional categories of COGs overrepresented in *Oleiphilus* as compared with other hydrocarbon-degrading *Oceanospirillales*. (For interpretation of the references to colour in this figure legend, the reader is referred to the web version of this article.)

We performed the analysis of distribution of Clusters of Orthologous Groups of proteins (COGs) in fully assembled genomes of oil-degrading *Oceanospirillales*. Interestingly, in *O. messinensis*, only 67.5% of all proteins were attributed to COGs, whereas in other marine alkane-degraders this value was in the range of 72–83% with *Alcanivorax*, the organism with the most streamlined genome having > 83% proteins in COGs. Unsurprisingly therefore that *Oleiphilus* had the largest percentage of proteins in the category R (general function prediction only) (Fig. 6). Likely, due to its largest genome size among analyzed genomes, the percentage of proteins of categories C and E (energy production and conversion and amino acid transport and metabolism, correspondingly) were underrepresented. As discussed above, the absolute number of mobile genetic elements (functional category X) in *Oleiphilus* is remarkably high, with 142, by far outperforming next-following *Oleispira antarctica* with just 53 active or inactivated transposases in the genome of the latter. This may be a result of *Oleiphilus* spp. being native inhabitants of the marine sediment, where they inevitably comes into a physical contact with other microorganisms, which is essential for the DNA acquisition/lateral gene transfer.

The census of proteins important in the signal transduction (in particular, with the domains HisK, MCP, GGDEF, GGDEF + EAL, EAL, HD-GYP, AC1, AC3, STYK, ABC1, PP2C and RRs) has revealed *O. messinensis* having by far the largest number of these proteins (345) with *Oleispira antarctica* (the runner-up) having just 170. This is also reflected in the Fig. 6, which shows the signal-transduction-related proteins having a highest percentage in total *in silico* proteomes of alkane-degrading *Oceanospirillales* (combined categories T + TK + KT + NT). This suggests that *Oleiphilus* may have a very sophisticated means for sensing the environment and react to the changes therein. This again points at the origin of *Oleiphilus* spp. from marine sediments, where the spatial environmental (physico-chemical) patchiness and heterogeneity are much more pronounced than in a relatively homogenous and constant water column.

## 3 Conclusion

The recent blowout of Deepwater Horizon platform in the Gulf of Mexico was followed by one of the largest offshore oil spills with ~ 4 million barrels crude oil released into the sea. Countless studies have reported a number of autochtonous marine hydrocarbon-degrading bacteria from the order *Oceanospirillales* have been identified to be actively involved in the oil degradation. The bloom of these organisms resulted in the rapid degradation of many oil constituents, hence highlighting the importance of OHCB in bioremediation of marine environment and a necessity of comprehensive studies to unveil the genomic and physiological backgrounds of hydrocarbonoclastic lifestyle of OHCB.

Despite having the largest genome among OHCB, *O. messinensis* exhibits a very narrow substrate profile and contains the largest numbers of mobile genetic elements, as compared with more streamlined genomes of other OHCB counterparts. With this study, we extended the list of OHCB whose genomes were fully sequenced to further expand our understanding of the efficiency and functional redundancy in hydrocarbon utilization by OHCB, the metabolic routes underlying their special hydrocarbon diet and their ecological success.

## Conflicts of interests

Authors declare no conflicts of interests.

## Appendix A Supplementary data

Supplementary material Image 1
