# Supplementary material for: The genome analysis of Oleiphilus messinensis ME102 (DSM 13489T) reveals backgrounds of its obligate alkane-devouring marine lifestyle
Source: Mar Genomics. 2017 Dec;36:41–7. doi: 10.1016/j.margen.2017.07.005 (PMC5847120; doi:10.1016/j.margen.2017.07.005)
Supplement: Supplementary file 1 — Supplementary material [file mmc1.docx]

**Supplementary information.**

**1. Amino acid identity between *Hahella chejuensis* KCTC 2396 and *Oleiphilus messinensis* ME102.**

Amino acid identity was calculated with ANI calculator (<http://enve-omics.ce.gatech.edu/aai/index> ; Rodriguez-R & Konstantinidis, 2016 ).

One-way AAI 1: 42.82% (SD: 16.65%), from 6286 proteins.

One-way AAI 2: 44.69% (SD: 16.93%), from 5547 proteins.

Two-way AAI: 53.17% (SD: 17.02%), from 3054 proteins.

**Supplementary Figure 1.** Amino acid identity between *Hahella chejuensis* KCTC 2396 and *Oleiphilus messinensis* ME102.


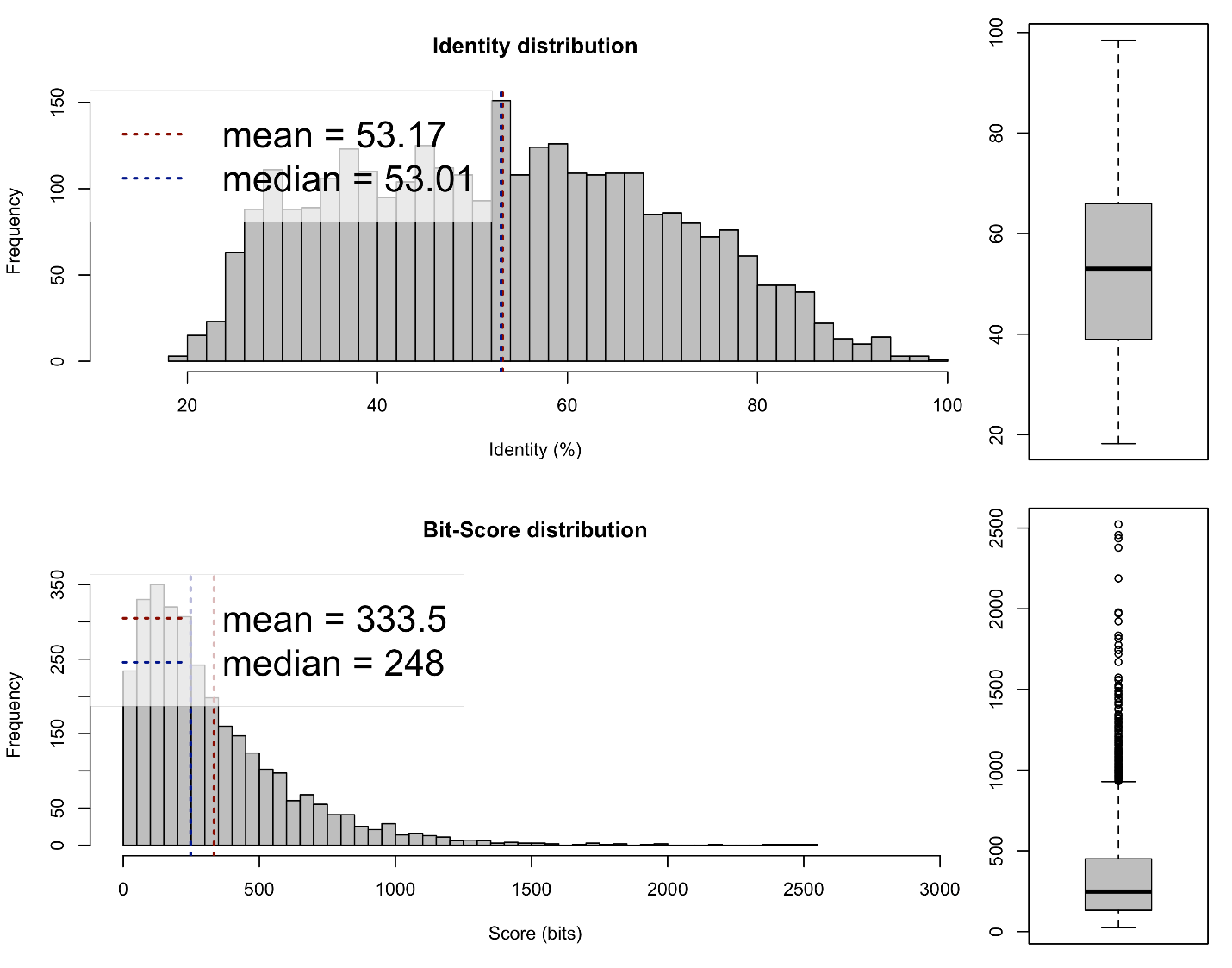


**2. Supplementary Figure 2. Maximum-likelihood phylogenetic tree of complete genomes of free-living gammaproteobacteria, taxonomically related to OMHCB based on concatenated alignment of 22 ribosomal proteins**


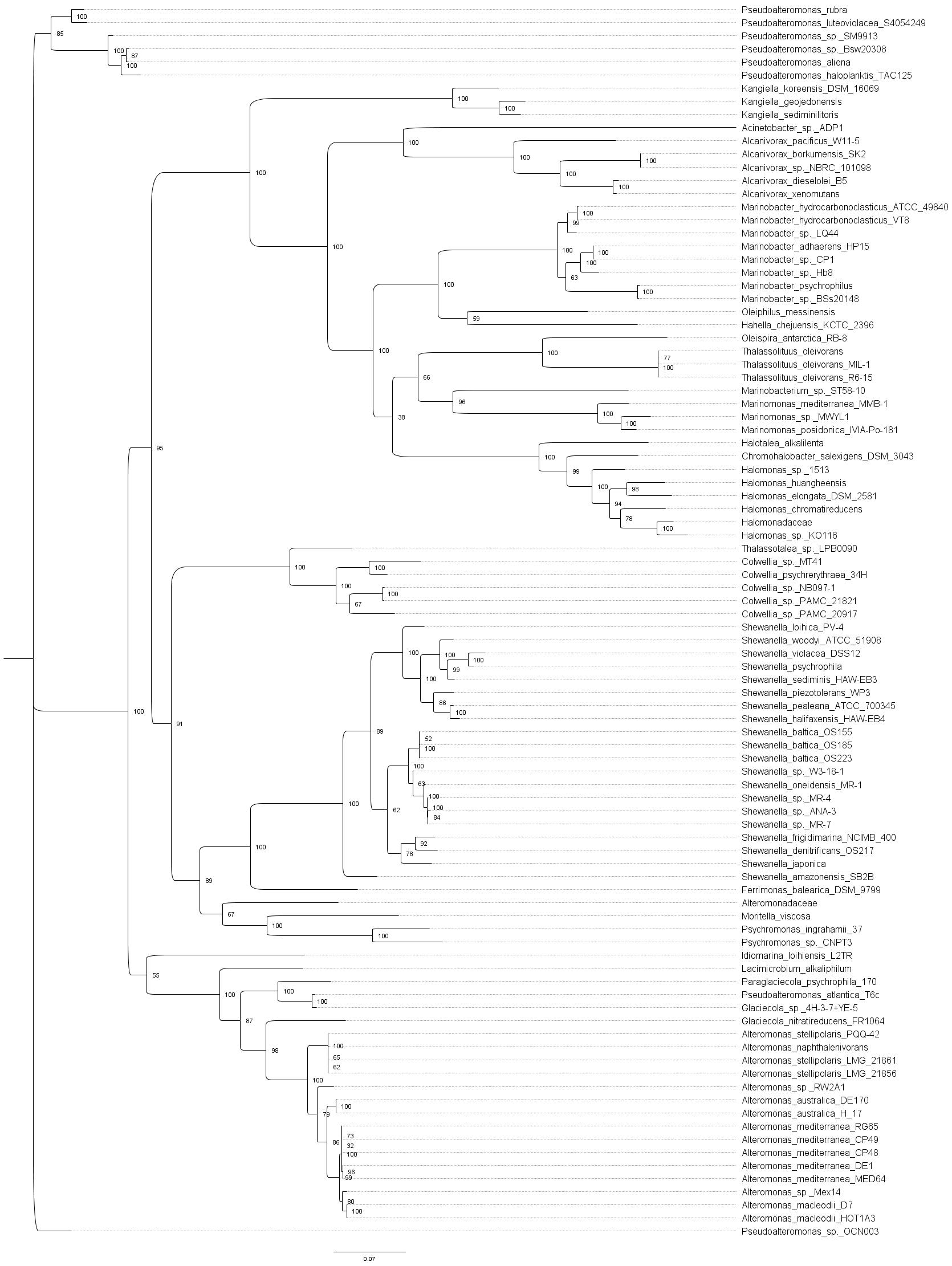


Supplementary Table 1. Mobile elements of *O.messinensis* ME_102

| **IS Family** | **ORFs Distribution*** | **Different IS(s)** | **Total IS(s)** |
| --- | --- | --- | --- |
| **IS4_ssgr_IS4** | **[2 / 0 / 0 / 0]** | **1** | **2** |
| **IS1595_ssgr_ISPna2** | **[0 / 1 / 0 / 0]** | **1** | **1** |
| **IS200_IS605_ssgr_IS1341** | **[0 / 1 / 0 / 1]** | **2** | **2** |
| **ISL3** | **[2 / 0 / 0 / 3]** | **5** | **5** |
| **IS1595_ssgr_IS1595** | **[2 / 0 / 0 / 0]** | **1** | **2** |
| **IS200_IS605_ssgr_IS605** | **[0 / 1 / 0 / 0]** | **1** | **1** |
| **IS5_ssgr_IS427** | **[2 / 1 / 0 / 0]** | **1** | **3** |
| **IS5_ssgr_IS5** | **[3 / 2 / 0 / 0]** | **1** | **5** |
| **IS3_ssgr_IS3** | **[17 / 6 / 0 / 0]** | **6** | **13** |
| **IS4_ssgr_ISH8** | **[12 / 1 / 0 / 3]** | **2** | **16** |
| **IS607** | **[1 / 0 / 0 / 0]** | **1** | **1** |
| **IS200_IS605_ssgr_IS200** | **[3 / 1 / 0 / 1]** | **3** | **5** |
| **ISNCY_ssgr_IS1202** | **[1 / 0 / 0 / 0]** | **1** | **1** |
| **IS256** | **[0 / 2 / 0 / 0]** | **2** | **2** |
| **IS66** | **[12 / 4 / 0 / 0]** | **4** | **9** |
| **IS200_IS605** | **[4 / 0 / 0 / 1]** | **3** | **5** |
| **ISAs1** | **[0 / 0 / 0 / 4]** | **1** | **4** |
| **Tn3** | **[0 / 0 / 0 / 1]** | **1** | **1** |
| **IS1182** | **[3 / 2 / 0 / 3]** | **3** | **8** |
| **IS701** | **[0 / 0 / 0 / 7]** | **1** | **7** |
| **IS630** | **[0 / 2 / 0 / 0]** | **2** | **2** |
| **IS1380** | **[18 / 6 / 0 / 0]** | **3** | **24** |
| **IS110_ssgr_IS1111** | **[3 / 0 / 0 / 0]** | **1** | **3** |
| **IS110** | **[0 / 3 / 0 / 0]** | **1** | **3** |

**^*^Legend: [Complete / Partial / Pseudogene / unknown]**

Supplementary Table 2. Distribution of COG functional categories in OMHCB.

| FC | *Alcanivorax borkumensis* | *Marinobacter hydrocarbono-clasticus* | *Oleispira antarctica* | *Thalassolituus oleivorans* | *Oleiphilus messinensis* |
| --- | --- | --- | --- | --- | --- |
| *C, Energy production and conversion* | 6,61 | 5,81 | 5,91 | 5,43 | 4,93 |
| *D, Cell cycle control, cell division, chromosome partitioning* | 1,39 | 1,14 | 1,11 | 1,12 | 1,08 |
| *E, Amino acid transport and metabolism* | 5,98 | 6,50 | 5,66 | **4,96** | **4,81** |
| *F, Nucleotide transport and metabolism* | 2,05 | 1,51 | 1,89 | 2,01 | 1,54 |
| *G, Carbohydrate transport and metabolism* | 2,57 | 2,96 | 2,03 | 2,36 | 2,22 |
| *H, Coenzyme transport and metabolism* | 4,23 | 3,39 | 4,13 | 3,65 | 3,24 |
| *I, Lipid transport and metabolism* | 5,16 | 3,72 | 3,68 | 3,82 | 3,98 |
| *J, Translation, ribosomal structure and biogenesis* | 7,37 | 5,70 | 6,14 | 6,65 | **4,95** |
| *K, Transcription* | 4,32 | 4,56 | 4,80 | 4,69 | 5,21 |
| *L, Replication, recombination and repair* | 3,74 | 3,11 | 3,02 | 3,10 | 2,48 |
| *M, Cell wall/membrane biogenesis;* | 5,89 | 4,90 | 4,56 | 4,66 | 4,57 |
| *N, Cell motility* | 0,42 | 1,30 | 1,17 | 1,14 | 1,38 |
| *O, Posttranslational modification, protein turnover, chaperones* | 4,59 | 3,97 | 3,95 | 4,14 | 3,58 |
| *P, Inorganic ion transport and metabolism* | 5,80 | 5,49 | 5,23 | 5,48 | 4,44 |
| *Q, Secondary metabolites biosynthesis, transport and catabolism* | 1,48 | 1,25 | **0,81** | **0,60** | 1,57 |
| *R, General function prediction only* | 7,04 | 6,18 | 6,56 | 6,43 | 7,37 |
| *S, Function unknown* | 5,65 | 4,39 | 4,87 | 5,51 | 5,00 |
| *T, Signal transduction mechanisms* | 9,90 | 16,76 | 16,22 | 15,60 | 12,91 |
| *U, Intracellular trafficking and secretion* | 1,06 | 1,34 | 1,51 | 1,74 | 1,50 |
| *V, Defense mechanisms* | 1,54 | 1,47 | 1,96 | 1,69 | 1,63 |
| *X, mobile genes* | **0,51** | **0,41** | 1,26 | 1,91 | 2,33 |
| *T+TK+KT+NT,*  *Signal transduction* | **13,29** | 22,47 | 21,81 | 20,94 | 22,84 |
| *Total proteins* | 2755 | 3804 | 3919 | 3662 | 5502 |
| *Proteins in COGs, %* | 2291 | 2902 | 2837 | 2654 | 3713 |
| *Proteins in COGs, %* | **83,16** | 76,29 | 72,39 | 72,47 | 67,48 |

**Supplementary references**

1. Rodriguez-R LM, Konstantinidis KT. (2016) The enveomics collection: a toolbox for specialized analyses of microbial genomes and metagenomes. PeerJ Preprints 4:e1900v1 https://doi.org/10.7287/peerj.preprints.1900v1
